# Safety and Efficacy of the Rho-Kinase Inhibitor (Ripasudil) in Bleb Needling after Trabeculectomy: A Prospective Multicenter Study

**DOI:** 10.3390/jcm13010075

**Published:** 2023-12-22

**Authors:** Yu Mizuno, Kaori Komatsu, Kana Tokumo, Naoki Okada, Hiromitsu Onoe, Hideaki Okumichi, Kazuyuki Hirooka, Gaku Aoki, Yukiko Miura, Yoshiaki Kiuchi

**Affiliations:** 1 Department of Ophthalmology and Visual Science, Hiroshima University, 1-2-3 Kasumi Minamiku, Hiroshima 734-8551, Japan; 2 Department of Biostatistics, Clinical Research Center, Hiroshima University Hospital, 1-2-3 Kasumi Minamiku, Hiroshima 734-8551, Japan; 3 Hiroshima Eye Clinic, 13-4, Noborimachi Nakaku, Hiroshima 730-0016, Japan

**Keywords:** glaucoma, trabeculectomy, needling, a rho-associated protein kinase inhibitor, ripasudil

## Abstract

Ripasudil, a rho-associated protein kinase inhibitor ophthalmic solution, shows a protective effect in preventing excessive scarring in vitro. This study aims to evaluate the safety and efficacy of ripasudil for glaucoma patients submitted to the needling procedure. In this prospective, multicenter, single-arm study, we included 20 eyes of 20 patients with glaucoma who underwent the needling procedure without antimetabolites. All patients administered ripasudil after needling for three months. The primary endpoint of this study was the safety of ripasudil in patients, and the secondary endpoint was the change in IOP at 12 weeks after the needling procedure. No serious complications were found in the patients. One eye experienced pruritus and conjunctival follicle, while another eye had conjunctival follicle. These complications were transient and resolved quickly after discontinuation of ripasudil. The mean preoperative IOP was 14.6 ± 4.6 mmHg, which decreased to 11.0 ± 4.7 mmHg (*p* = 0.0062) at 1 week postoperatively. The IOP reduction effect continued to 12 weeks (11.8 ± 3.1 mmHg; *p* = 0.0448). The administration of the ROCK inhibitor, ripasudil, after the needling procedure is safe and effective in maintaining IOP for 12 weeks.

## 1. Introduction

Glaucoma is an optic neuropathy characterized by gradual, progressive morphological changes in the optic disc and visual field loss [1]. Trabeculectomy (TLE) is a traditional surgical technique for lowering intraocular pressure (IOP) with long-term efficacy in slowing the advancement of visual field loss among patients with glaucoma [2]. However, over time, we often experience a loss of IOP control. When filtration fails after the initial success of trabeculectomy, the most common cause is fibrosis, a natural part of the wound healing process.

The transconjunctival bleb needling procedure is designed to address the inadequacies of failing blebs thorough the mechanical removal of adhesions. Failed blebs often require multiple needling procedures due to fibrosis.

Ripasudil (Glanatec^®^, ophthalmic solution 0.4%, Kowa Company, Ltd., Tokyo, Japan) is an approved ophthalmic solution in Japan for managing glaucoma or ocular hypertension, which received approval in 2014. Several reports indicate that topical instillation treatment with the ROCK inhibitors Y-27632 [3,4] and ripasudil [5] can mitigate excessive scarring in vitro and following glaucoma filtration surgery in mouse or rabbit models. In our previous study, we reported that administration of ripasudil following the needling procedure with mitomycin C (MMC) did not show a more significant reduction in IOP when compared to the MMC needling procedure alone. However, this study suggests that ripasudil might reduce the number of IOP-lowering agents required after the needling procedure [6]. It is worth noting that this study was retrospective, and all subjects had received MMC just before needling. The antifibrosis effect of MMC might mask the effect of ripasudil.

In the present study, we conducted a prospective assessment of the safety profile in glaucoma patients who underwent needling without MMC and subsequently received ripasudil following the procedure. The study also thoroughly investigated the effectiveness of ripasudil administration in preventing bleb failure by suppressing fibrosis within the bleb.

## 2. Materials and Methods

In this prospective, multicenter, open-label, single-arm, phase II study, we evaluated patients at Hiroshima University Hospital and Hiroshima Eye Clinic in Japan. The study was conducted in accordance with the Declaration of Helsinki and received approval from the Hiroshima University Certified Review Board (Approval No. CRB210008, 26 May 2022). We analyzed 20 eyes of 20 glaucoma patients who underwent the needling procedure without the use of antimetabolites. Clinical signs of scarring determined by glaucoma specialists, including increased IOP following TLE and vascularization of the bleb, were the main indicators for the needling procedure. During the needling procedure, none of the patients encountered intraoperative complications. Our study specifically enrolled patients who underwent the needling procedure at least three months after TLE and excluded individuals with a previous history of conjunctival surgery (except for TLE).

All patients underwent comprehensive ophthalmic examination, and the following data were collected: sex, age, type of glaucoma, IOPs (Goldmann Applanation Tonometer, Haag-Streit, Köniz, Switzerland), lens status, the time interval since the most recent TLE, number of antiglaucoma medications, and corneal endothelial cell density. To categorize the morphological appearance observed through slit lamp examination, we assessed the characteristics of the blebs utilizing the Indiana Bleb Appearance Grading Scale. The scale takes into account the parameters of height, extent, vascularity, and leakage, which is graded through the Seidel test [7].

The primary endpoint of this study was to evaluate the safety profile of ripasudil administrated promptly in patients who underwent the needling procedure. Ophthalmic and systemic evaluations were conducted to scrutinize the occurrence of local and systemic adverse events (AEs) as well as adverse drug reactions (ADRs). AEs were documented irrespective of whether abnormalities occurred in the contralateral eye to which the ripasudil ophthalmic solution was administered. 

We also assessed the treatment’s efficacy as the secondary endpoint. We characterized absolute success as achieving a >20% reduction in IOP from the preneedling baseline without the use of antihypertensive medications (excluding the instillation of ripasudil). If the IOP exceeded the predefined criteria in two consecutive measurements, failure was deemed to have occurred at the initial time point when the IOP first exceeded the criteria. The necessity for repeat needling or an alternative glaucoma surgical intervention was categorized as a failure. We further characterized relative success as achieving an IOP within the ranges (A) 4 mmHg or higher but less than 22 mmHg, (B) 4 mmHg or higher but less than 19 mmHg, (C) 4 mmHg or higher but less than 16 mmHg, or (D) 4 mmHg or higher but less than 13 mmHg, in an attempt to adhere to the outcome criteria outlined by the World Glaucoma Association guidelines. If the IOP exceeded the defined criteria in two consecutive measurements, failure was deemed to have occurred at the initial time point when the IOP first exceeded the above criteria. The necessity for repeat needling or another glaucoma surgical intervention was classified as a treatment failure.

### 2.1. Needling Procedure

All needling procedures were conducted in an outpatient department by glaucoma specialists listed in the protocol. Following the application of topical anesthesia (0.4% oxybuprocaine and 0.1% adrenaline), iodine and polyvinyl alcohol (PA·IODO ophthalmic and eye-washing solution, diluted six times with saline solution) were administered to the external eye. Subsequently, a 27-gauge needle was used to inject 0.1 mL of 2% xylocaine with epinephrine approximately 10 mm distal to the bleb. The needle was introduced between the conjunctiva and sclera, and fibrotic tissues were incised and elevated. After the procedure, patients were instructed to use ripasudil twice daily for three months, in conjunction with topical antibiotics (1.5% levofloxacin) and an anti-inflammatory (0.1% fluorometholone ophthalmic suspension) ophthalmic solution three times daily for one week.

### 2.2. Statistical Analysis

All the original data collected were stored in the electronic data capture (EDC) system (REDCap 12.0.33, Vanderbilt University). The EDC system was digitally secured on a password-protected internet server, and the investigators from the study team entered the data directly into the EDC system. 

Considering the exploratory nature of this study, the sample size was projected by referencing historical medical records, which included an assessment of the annual patient volume at both Hiroshima University Hospital and Hiroshima Eye Clinic.

The analysis sets for this study were delineated as the Full Analysis Set (FAS) and the Per-Protocol Set (PPS). The FAS encompassed all participants in the study, excluding instances that failed to meet the eligibility criteria. The PPS was derived from the FAS, with the exclusion of individuals lacking available measurements for the primary endpoints and patients with substantial deviations from the study protocol. In this investigation, the FAS and PPS were congruent. 

The primary endpoint of this study, focusing on the safety of administering ripasudil immediately following the needling procedure, was evaluated by quantifying the incidence of AEs and adverse drug reactions (ADRs), expressed in both absolute numbers and percentages. The analysis population encompassed all patients who received a minimum of one dose of ripasudil, and they were categorized based on the nature of AEs or ADRs.

For the secondary endpoint, aimed at assessing the efficacy of ripasudil, the results underwent analysis utilizing either Student’s *t*-test or Wilcoxon’s rank sum test. Survival rates were estimated by employing the Kaplan–Meier method. All statistical analyses were conducted using SAS version 9.4 (SAS Institute Inc., Cary, NC, USA). Measurement data are presented as mean ± standard deviation with a 95% confidence interval. Statistical significance was considered when the *p*-value was <0.05.

## 3. Results

We conducted an analysis on 20 eyes belonging to 20 glaucoma patients who underwent bleb needling without MMC due to TLE failure with a fornix-based conjunctival flap using MMC. A summary of demographic data is provided in Table 1. All patients were Japanese. There were 12 males and 8 females, with a mean age of 70.3 ± 11.8 years old (range: 49–89 years old). The mean time from TLE to needling was 4.2 ± 6.7 years (range: 0.30–30.02 years).

During the study period, no serious AEs or ADRs were found in the patients. We had two minor ADRs: one eye had pruritus and conjunctival follicle, and one eye had conjunctival follicle. However, the complications were transient and resolved quickly after the discontinuation of ripasudil (Table 2). 

**Figure 1:**
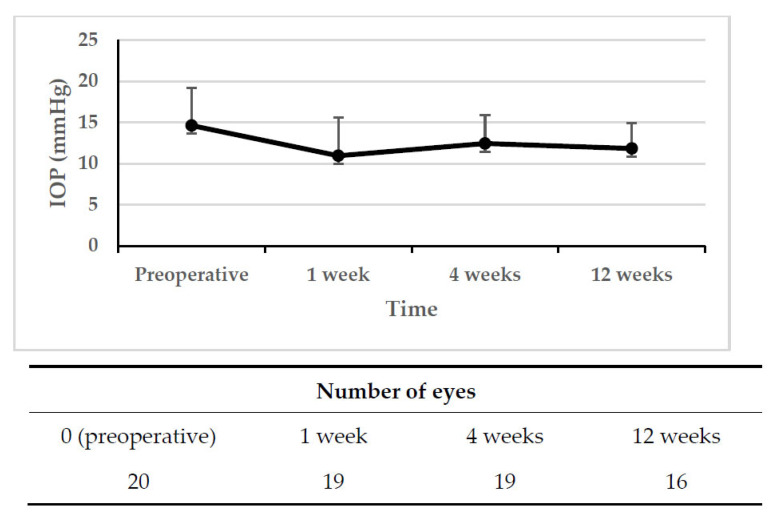
Preoperative and postoperative mean IOPs (mmHg). Error bars show standard deviation. The mean preoperative IOP was lowered immediately after the needling procedure (*p* = 0.0006, Wilcoxon’s rank sum test). The effect of IOP reduction was continued to 12 weeks (*p* = 0.0012, Wilcoxon’s rank sum test). Error bars show the standard deviation.

**Figure 2:**
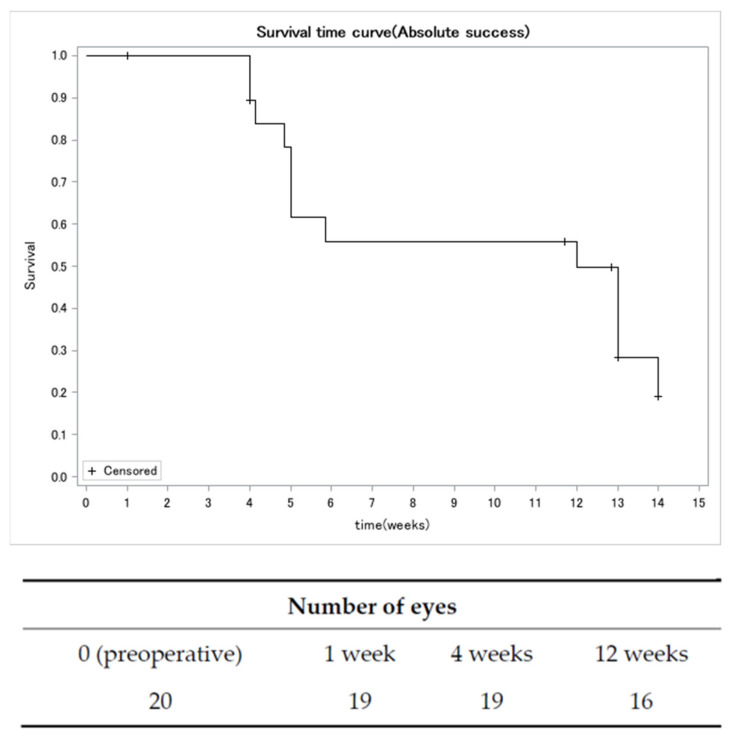
Kaplan–Meier survival plot for absolute success. We characterized absolute success as achieving a >20% reduction in IOP from the preneedling baseline without the use of antihypertensive medications (excluding the instillation of ripasudil). If the IOP exceeded the predefined criteria in two consecutive measurements, failure was deemed to have occurred at the initial time point when the IOP first exceeded the criteria. The necessity for repeat needling or an alternative glaucoma surgical intervention was categorized as a failure.

**Figure 3:**
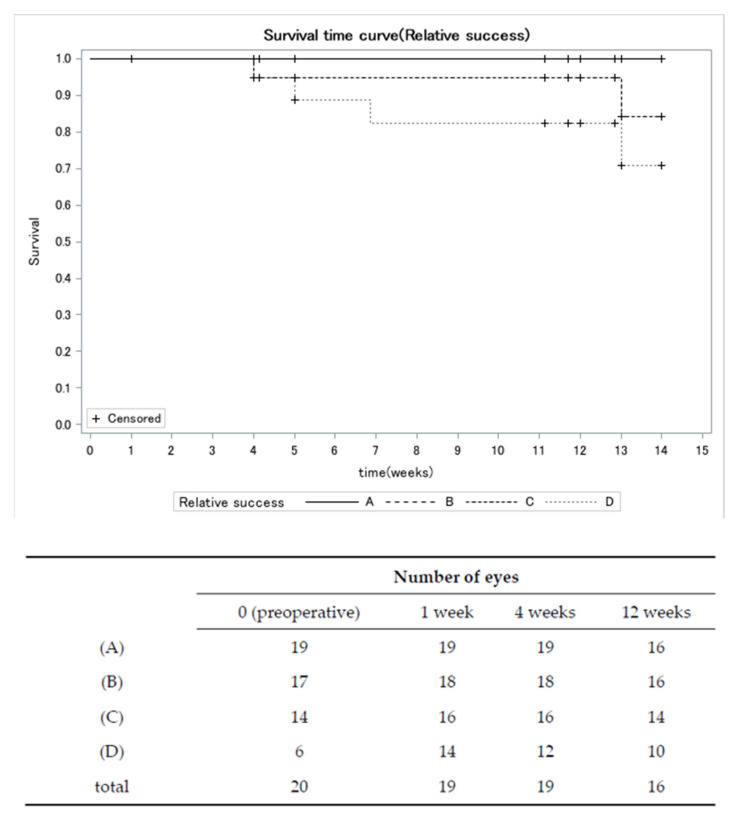
Kaplan–Meier survival plot for relative success. We characterized relative success as achieving an IOP within the following ranges: (A) 4 mmHg or higher but less than 22 mmHg, (B) 4 mmHg or higher but less than 19 mmHg, (C) 4 mmHg or higher but less than 16 mmHg, or (D) 4 mmHg or higher but less than 13 mmHg. If the IOP exceeded the defined criteria in two consecutive measurements, failure was deemed to have occurred at the initial time point when the IOP first exceeded the above criteria. The necessity for repeat needling or another glaucoma surgical intervention was classified as a treatment failure. Note: Survival rate and censoring of Relative success B were equal to Relative success A.

Figure 1 illustrates a significant reduction in the mean IOP. The mean preoperative IOP was 14.6 ± 4.6 mmHg, which decreased to 11.0 ± 4.7 mmHg (a reduction of 25.1%, *p* = 0.0006) at 1 week postoperatively. This reduction in IOP was maintained up to 12 weeks (11.8 ± 3.1 mmHg; a reduction of 19.1%, *p* = 0.0012) (Figure 1). The Kaplan–Meier survival plot for absolute success showed a 12-week survival rate of 50.0% (Figure 2). For criteria A, B, C, and D, the relative success rates were 100.0%, 100.0%, 95.0%, and 82.0%, respectively (Figure 3).

There were no significant differences between the preoperative and postoperative mean numbers of antiglaucoma medications at 12 weeks (*p* = 0.10) (Table 3). The number of eyes with antiglaucoma medications before the needling procedure was nine (45%). The most utilized type of antiglaucoma medication before the needling procedure was prostaglandins (PG), followed by beta-blockers (BB). The subsequent use included carbonic anhydrase inhibitors (CAI) and alpha-2-agonists (AA) (PG: two eyes; PG + BB: two eyes; PG + CAI: one eye; PG + BB + CAI: two eyes; PG + BB + CAI + AA: one eye; BB: one eye). The number of eyes with antiglaucoma medications other than ROCK inhibitors 12 weeks after the needling procedure was two (PG: one eye; BB: one eye).

The morphological slit lamp appearance of the blebs showed no significant difference between the preoperative and postoperative periods during the 12 weeks (Table 4).

Furthermore, there was no significant difference in corneal endothelial cell density preoperatively and postoperatively at 12 weeks (*p* = 0.14) (Table 5). 

## 4. Discussion

This study showed that ripasudil administration after needling without any antifibrotic agents, such as MMC or 5-FU, did not result in any serious AEs or ADRs. Hidenobu T. et al. documented the results of a phase I clinical trial, elucidating that the seven-day repeated instillation of ripasudil exhibited manageable adverse events (AEs). These included mild to moderate conjunctival hyperemia observed in the healthy volunteers, and such events were resolved within 90 min following instillation [8]. The safety profile of ripasudil exhibited similarity to that observed in patients diagnosed with ocular hypertension or open-angle glaucoma [9,10]. In this study, only two eyes had temporally pruritus and conjunctival follicle, and these were resolved quickly after the discontinuation of ripasudil. These results may indicate that the safety of ripasudil after glaucoma filtration surgery was equivalent to that in patients with ocular hypertension or glaucoma who underwent filtration surgery. 

The most efficacious treatment for patients with glaucoma is the reduction in IOP [11,12]. TLE is considered the gold-standard surgical technique for lowering the IOP [2]. This procedure establishes a drainage pathway connecting the anterior chamber and the sub-Tenon space, resulting in the formation of a filtrating bleb within the subconjunctival space for the aqueous humor. The success of TLE relies on the continuous passage of aqueous humor between the anterior chamber and subconjunctival space. Nevertheless, the primary factors contributing to procedure failure are commonly attributed to the proliferation of fibroblasts and the formation of scars at the conjunctival and episcleral interface of the filtrating bleb [13,14]. During glaucoma filtration surgery, the scleral flap or conjunctiva becomes exposed to cytokines and growth factors released by various types of inflammatory cells and fibroblasts [15]. These reactions elicit the activation and migration of fibroblasts and other inflammatory cells. Activated fibroblasts generate and secrete extracellular matrix, such as collagen, leading to fibrosis. ROCK inhibitors mitigate the expression of extracellular-matrix-degrading enzymes in fibroblasts, thereby reducing the secretion of extracellular matrix [16]. Ibrahim et al. revealed that the ROCK inhibitor Y-27632 has the potential to inhibit fibrosis and enhance outcomes after glaucoma filtration surgery. This is achieved by inhibiting the transdifferentiation of Tenon fibroblasts into myofibroblasts and transforming growth factor β (TGF-β) and mitogen-activated protein kinase signaling after surgery [3]. Ko et al. reported that ripasudil suppressed postoperative scar formation by altering the expression of α-SMA and vimentin after filtration surgery in a mouse model [5]. Honjyo M. et al. also reported that the topical instillation of Y-27632 inhibited wound healing and fibroproliferation after filtration surgery in a rabbit model by reducing the expression of α-SMA in the immunohistochemical staining of blebs postoperatively [4].

There are several treatment options available for addressing bleb failure after trabeculectomy, including antihypertensive medications, surgical treatments such as needling with or without antimetabolites like 5-FU or MMC [17,18,19], or surgical revisions with various techniques [20]. Currently, there is no universally accepted gold-standard treatment for bleb failure, and different glaucoma specialists may adapt their practices based on their individual experiences [21]. Transconjunctival bleb needling is designed to rejuvenate failing blebs by mechanically removing adhesions. To prevent fibroblast proliferation, 5-FU or MMC are commonly used in bleb needling procedures through subconjunctival injection [22,23,24]. The potential for achieving IOP control and reducing the need for antiglaucoma medications following needling surgery with antimetabolites is noteworthy [24]. Considering these, this study provides a prospective clinical assessment of administering the ROCK inhibitor ripasudil to clinically reduce subconjunctival scarring after the initial needling procedure without the subconjunctival injection of antifibrotic agents. This study demonstrates that ripasudil administration maintains IOP and survival rate after needling procedure for 12 weeks. Despite counting ripasudil itself as an antiglaucoma medication, there was no observed increase in the number of antiglaucoma medications from the preoperative period to 12 weeks after the needling procedure. In a prior study, we reported a reduction rate of 27.22% after the needling surgery with MMC subconjunctival injection, with IOP decreasing from 16.9 ± 4.5 mmHg to 12.3 ± 4.8 mmHg (*n* = 15) at 12 weeks post procedure [6]. A Kaplan–Meier survival plot is presented with the same definition as in this study, showing a 12-week survival rate of 60.00% after needling with MMC. While it may be challenging to make a direct comparison, ripasudil administration after needling appears to help maintain the filtration bleb through antifibrotic effects, without resulting in any serious complications.

The success of trabeculectomy or needling procedures largely depends on the formation of a filtration bleb. Bleb morphology is a crucial factor for success. Lee YS et al. identified that a smaller central bleb extension and flatter height are morphological risk factors for failure [25]. The needling procedure generally increased the spread of the bleb. In our study, although there was a tendency for improvement and maintenance of bleb extension and height after needling with the use of the ROCK inhibitor, there was no statistically significant difference in bleb extension and height before and after the needling procedure. While the study duration was too short to definitively assess the effects of the ROCK inhibitor on maintaining morphological bleb formation thorough its antifibrotic effects, it is possible that ripasudil administration after the needling procedure preserved the morphology of the bleb, potentially resulting in a reduction in IOP.

A major concern with the use of antimetabolites is their safety, as these were reported to be potentially toxic to ocular tissues. Helosa A. et al. reported that there was no significant difference in preoperative corneal endothelial cell density at 12 months [18]. However, several studies indicated that the subconjunctival injection of antimetabolites can cause severe damage to corneal endothelial cells [26,27,28,29]. While such complications are rare, they cannot be resolved through observation or treatment with ocular instillation and may require corneal transplantation. ROCK inhibitors were reported to have the potential to improve corneal endothelial diseases, such as Fuchs endothelial dystrophy or corneal edema, and to have a protective effect on corneal endothelial cells after cataract surgery [30,31,32,33,34]. The exact mechanism by which ROCK inhibitors improve corneal endothelial cells has not been fully elucidated, but it is possible that these promote the reactivation of cell proliferation and migration, and the restoration of the corneal endothelial pump and barrier functions, contributing to the regeneration of corneal endothelial cells [29,35]. In this study, there was no change in corneal endothelial cell density more than 12 weeks after the needling surgery. This result suggests that ripasudil may potentially preserve corneal endothelial cells after needling surgery by promoting the regeneration of corneal endothelial cells.

This study has several limitations. The first is the lack of a dose–response test including the optical frequency of ripasudil or the optical ripasudil concentration. However, ripasudil, Glanatec^®^, was approved in Japan as an ophthalmic solution at a concentration of 0.4% with instillation two times per day. We believe that using the approved range is an important indicator of the safety of patients. Second, this study was designed as a single-arm trial because we defined safety as the primary endpoint. Therefore, the assessment of effectiveness involves comparing patients before and after needling procedures. Specifically, the efficacy of ripasudil cannot be adequately evaluated in terms of its effect on the overall course of the disease beyond its pharmacological action, which makes it an inadequate approach compared to a placebo-controlled design. Kim JS et al. reported that bleb needling procedures without any antimetabolites resulted in an IOP reduction over 12 weeks (from 23.0 ± 6.2 mmHg to 16.4 ± 5.7 mmHg, *n* = 72) [36]. While it is challenging to make a direct comparison and the follow-up period was too short to calculate survival, our findings suggest that administration of ripasudil after the needling procedure may be more effective for lowering and maintaining IOP than not using any antimetabolites, even though the preneedling IOP was lower than their result (from 14.6 ± 4.6 mmHg to 11.8 ± 3.1 mmHg, *n* = 20). This may be attributed to the antifibrosis effect of ripasudil. Since there are no previous reports on the clinical use of ripasudil after needling without using antimetabolites such as MMC or 5-FU, it is difficult to predefine the obvious criteria of efficacy in this study. Therefore, despite the limitations in controlling bias, we plan to use the results of this study’s efficacy as a reference for future studies. The study will consider success and failure criteria related to reductions in the number of preprocedure antiglaucoma medications. We also plan to incorporate a control group to assess the potential IOP-lowering effect of ripasudil due to its antifibrotic action. 

## 5. Conclusions

In conclusion, ripasudil administration after needling is safe and effective for controlling IOP, and it seems to have a potential role in maintaining corneal endothelial cells.

## Figures and Tables

**Table 1:** Demographic characteristics.

Total number of eyes	20
Mean preoperative IOP (mmHg)	14.6 ± 4.6
Mean number of antihypertensive medications	1.0 ± 1.3
Age (years)	70.3 ± 11.8
Gender (male/female)	12/8
Laterality (right/left)	9/11
Glaucoma classification	
POAG	18
PACG	0
Secondary glaucoma	2
Time from TLE surgery to needling (Y)	4.3 ± 6.7
Lens status	
Phakia	7
IOL	13

IOP, intraocular pressure; POAG, primary open-angle glaucoma; PACG, primary angle-closure glaucoma; TLE, trabeculectomy.

**Table 2:** Type of side effects.

Total number of side effects	2 (10.0%)
Type of side effects	
conjunctival follicle	2 (10.0%)
pruritus	1 (5.0%)

Note: There was one eye with pruritus and conjunctival follicle concurrently manifested in the same eye.

**Table 3:** Number of antiglaucoma medications.

	Number of Antiglaucoma Medications	*p* *
preoperative	0.95 ± 1.28	
1 week	1.00 ± 0.00	0.14
4 weeks	1.05 ± 0.23	0.12
12 weeks	1.19 ± 0.54	0.10

* Wilcoxon’s rank sum test.

**Table 4:** Changes in the morphological slit appearance of the blebs.

Indiana Bleb Appearance Grading Scale, Height (H0–H3) **									
		Preoperative							
		H0	H1		H2		H3	Total (%)	p *
1 weeks	H0	2	0		1		0	3 (15%)	0.23
	H1	1	3		0		0	4 (20%)	
	H2	0	5		4		0	9 (45%)	
	H3	0	0		1		2	3 (15%)	
	missing values	1	0		0		0	1 (5%)	
	Total (%)	4 (20%)	8 (40%)		6 (30%)		2 (10%)	20 (100%)	
4 weeks	H0	2	0		0		9	2 (11%)	0.15
	H1	2	3		1		0	6 (32%)	
	H2	0	3		4		1	8 (42%)	
	H3	0	1		1		1	3 (16%)	
	missing values	0	0		0		0	0 (0%)	
	Total (%)	4 (21%)	7 (37%)		6 (32%)		2 (11%)	19 (100%)	
12 weeks	H0	2	0		0		0	2 (13%)	0.07
	H1	1	2		1		0	4 (25%)	
	H2	1	4		2		1	8 (50%)	
	H3	0	1		1		0	2 (13%)	
	missing values	0	0		0		0	0 (0%)	
	Total (%)	4 (25%)	7 (44%)		4 (25%)		1 (6%)	16 (100%)	
Indiana Bleb Appearance Grading Scale, Extent (E0–E3) ***									
		Preoperative							
		E0	E1		E2		E3	Total (%)	p *
1 weeks	E0	3	0		0		0	3 (15%)	0.06
	E1	0	7		0		0	7 (35%)	
	E2	1	3		4		0	8 (40%)	
	E3	0	0		1		0	1 (5%)	
	missing values	1	0		0		0	1 (5%)	
	Total (%)	5 (25%)	10 (50%)		5 (25%)		0 (0%)	20 (100%)	
4 weeks	E0	3	0		0		0	3 (16%)	0.06
	E1	1	7		0		0	8 (42%)	
	E2	1	2		4		0	7 (37%)	
	E3	0	0		1		0	1 (5%)	
	missing values	0	0		0		0	0 (0%)	
	Total (%)	5 (26%)	9 (47%)		5 (26%)		0 (0%)	19 (100%)	
12 weeks	E0	3	0		0		0	3 (19%)	0.06
	E1	1	5		0		0	6 (38%)	
	E2	1	3		3		0	7 (44%)	
	E3	0	0		0		0	0 (0%)	
	missing values	0	0		0		0	0 (0%)	
	Total (%)	5 (31%)	8 (50%)		3 (19%)		0 (0%)	16 (100%)	
Indiana Bleb Appearance Grading Scale, Vascularity (V0–V4) ****									
		Preoperative							
		V0	V1	V2		V3	V4	Total (%)	p *
1 weeks	V0	1	0	0		0	0	1 (5%)	0.81
	V1	0	3	0		0	0	4 (20%)	
	V2	1	0	1		2	0	7 (35%)	
	V3	1	0	4		6	0	8 (35%)	
	V4	0	0	0		0	0	0 (0%)	
	missing values	0	0	0		1	0	1 (5%)	
	Total (%)	3 (15%)	3 (15%)	5 (25%)		9 (45%)	0 (0%)	20 (100%)	
4 weeks	V0	1	0	0		0	0	1 (5%)	1.00
	V1	0	3	1		0	0	4 (21%)	
	V2	1	0	4		1	0	6 (32%)	
	V3	0	0	0		8	0	8 (42%)	
	V4	0	0	0		0	0	0 (0%)	
	missing values	0	0	0		0	0	0 (0%)	
	Total (%)	2 (11%)	3 (16%)	5 (26%)		9 (47%)	0 (0%)	19 (100%)	
12 weeks	V0	0	0	0		0	0	0 (0%)	1.00
	V1	0	2	0		0	0	2 (13%)	
	V2	1	0	4		1	0	6 (38%)	
	V3	0	0	0		8	0	8 (50%)	
	V4	0	0	0		0	0	0 (0%)	
	missing values	0	0	0		0	0	0 (0%)	
	Total (%)	1 (6%)	2 (13%)	4 (25%)		9 (56%)	0 (0%)	16 (100%)	
Indiana Bleb Appearance Grading Scale, Leakage (S0–S2) *****									
		Preoperative							
		S0	S1				S2	Total (%)	p *
1 weeks	S0	19	0				0	19 (95%)	
	S1	0	0				0	0 (0%)	
	S2	0	0				0	0 (0%)	
	missing values	1	0				0	1 (5%)	
	Total (%)	20 (100%)	0 (0%)				0 (0%)	20 (100%)	
4 weeks	S0	19	0				0	19 (100%)	
	S1	0	0				0	0 (0%)	
	S2	0	0				0	0 (0%)	
	missing values		0				0	0 (0%)	
	Total (%)	19 (100%)	0 (0%)				0 (0%)	19 (100%)	
12 weeks	S0	19	0				0	16 (100%)	
	S1	0	0				0	0 (0%)	
	S2	0	0				0	0 (0%)	
	missing values	1	0				0	0 (0%)	
	Total (%)	16 (100%)	0 (0%)				0 (0%)	16 (100%)	

* Wilcoxon’s rank sum test. ** Height: H0, flat bleb without visible elevation; H1, low bleb elevation; H2, moderate bleb elevation; and H3, high bleb. *** Extent: E0, no visible bleb extent equal to less than 1 clock hour; E1, extent equal to or greater than 1 clock hour but less than 2 clock hours; E2, extent equal to or greater than 2 clock hours but less than 4 clock hours; and E3, extent equal to or greater than 4 clock hours. **** Vascularity: V0, avascular/white (no microcysts visible on slit lamp examination); V1, avascular/cystic (microcysts of the conjunctiva visible on slit lamp examination); V2, mild vascularity; V3, moderate vascularity; and V4, extensive vascularity (vascular engorgement). ***** Leakage: S0, no bleb leak; S1, pinpoint transconjunctival leakage visible on the bleb surface (at multiple points), without streaming of fluid within 5 s of application; and S2, streaming aqueous egress visible within 5 s of application of fluorescein.

**Table 5:** Changes in corneal endothelial cell density.

	Cells/mm^2^	*p* *
Preoperative	2422.24 ± 479.54	
1 week	2238.34 ± 479.75	0.06
4 weeks	2375.89 ± 409.54	0.02
12 weeks	2269.53 ± 436.34	0.14

* Wilcoxon’s rank sum test.

## Data Availability

Data are contained within the article.
